# Challenges of clinical accompaniment amongst undergraduate nursing students: University of KwaZulu-Natal

**DOI:** 10.4102/hsag.v29i0.2535

**Published:** 2024-07-05

**Authors:** Seaka Ramoeletsi, Boikhutso Tlou

**Affiliations:** 1 Department of Public Health, Discipline of Public Health Medicine, University of KwaZulu-Natal, Durban, South Africa

**Keywords:** clinical accompaniment, clinical learning practice, facilitation, challenges, student nurses, clinical setting, clinical supervision, clinical learning environment

## Abstract

**Background:**

Clinical accompaniment is an activity predominantly supervised by the clinical facilitator to develop the skills of the students. In South Africa, clinical accompaniment aims to develop the skills of the students to equip them in delivering efficient health services to the patients. Previous studies revealed that students experienced challenges and were negatively affected due to inadequate clinical accompaniment in the learning practice.

**Aim:**

The aim was to determine the challenges faced by University of KwaZulu-Natal (UKZN) undergraduate nursing students during their clinical accompaniment.

**Methods:**

An observational cross-sectional study design, with an analytic component was implemented. Questionnaires were used to collect data. Of the 400 registered nursing students, 245 were undergraduates; of these, 241 consented to participate in this study. Data captured into SPSS Statistics Package V28. ANOVA were used in comparing challenges amongst participants. A *p*-value less than 0.05 was considered significant.

**Results:**

A total of 241 participants responded to the questionnaires, which yielded a response rate of 98.4%. This study comprised first-year (32.4%), second-year (32.8%) and third-year (34.9%) students. There was no remarkable difference in terms of challenges amongst study participants (1st; 2nd; 3rd), *p*=0.592.

**Conclusion:**

This study revealed the challenges faced by undergraduate nursing students during their clinical accompaniment.

**Contribution:**

Study results might assist in developing effective guidelines to resolve the challenges encountered by students.

## Introduction

Nursing is a clinical based programme; hence, the necessity for teaching of nursing students in practice is critical (Heradien, 2019). Clinical accompaniment forms the cornerstone of nursing education and should therefore be considered by higher learning institutions as a vital component of the undergraduate nursing curriculum (Oermann, De Gagne & Phillips 2018). Clinical accompaniment is described as the direct supervision and practical support provided to the students by registered nurses (RNs) with the intention of increasing competence and confidence in the student. It is also described as a structured activity that is conducted by the clinical facilitator to enable student nurses to overcome their need for assistance and support, ensuring theory–practice integration (Van Graan & Williams, 2017). Furthermore, it is an integral and crucial component in facilitation of student nurses, as it guides structure of learning environment, offers professional support, provides feedback on student performance, and augments the students’ problem-solving skills.

Through effective clinical accompaniment, nursing students can practise what the profession requires and as a result become competent practitioners in the field. It is the duty of the RNs to account for and monitor the care students provide to the patients. Thus, RNs are obligated to teach, mentor, and guide students in clinical practice at their hospital wards in order to help students to care for and maintain the health of the patients (South African Nursing Council [SANC] 2013). Aktas and Karabulut (2016) determined that clinical accompaniment is provided to undergraduate nursing students with the aim of developing their professional skills and knowledge required in lifelong learning as well as critical thinking that will enable them to gain in self-confidence (Aktas & Karabulut 2016). Other scholars such as Kaphagawani and Useh (2013) citing Papastavrou et al. reported that the environment for learning should promote learning and provide the appropriate support for its skilled personnel. A clinical learning environment (CLE) that is rich in learning experiences but with inadequate support discourages the students from pursuing their goals and gaining experience. This lack of support most often will result in the loss of learning, growth, and experiences (Kaphagawani & Useh 2013).

Previous studies found that a CLE offering limited experiences but rich in support may offer opportunities for nursing students to explore new health needs of the patients (Mukumbang & Adejumo 2014). Moreover, other scholars such as Anagor et al. (2021) confirmed that the need for supervision of nursing students remains uppermost to ensure that accurate and correct information as well as appropriate skills are constantly employed and developed by the students. Experienced and knowledgeable clinical staff will assist in promoting the learning of students by providing an effective learning environment (Anagor et al. 2021). Needham, Mcmurray and Shaban (2016) in a study conducted, indicated that a well-planned clinical accompaniment programme provided an opportunity for the integration and application of theory, knowledge, and skills. Thus, this is crucial to developing applied and social skills required to become effective members of the health team. Some studies found that professional nurses were required to contribute to the growth of the profession of nursing through teaching, mentoring, and supporting the nursing students during clinical accompaniment (Jamshidi et al. 2016).

As discussed earlier, in South Africa, clinical accompaniment is mandatory for all nursing students while on training. Training for nurses is regulated by the South African Nursing Council (SANC) by stipulating the needed theory and practice standards (SANC 2013). All nursing students are required to register with the SANC after enrolment at a nursing education institution, and theoretical practice must be carried out at institutions. Facilitation is mandatory at hospitals and clinics as SANC maintains that the overall purpose of clinical accompaniment is to prepare students according to the level of training. This in turn ensures that on completion of the course, students are able to demonstrate the ability to resolve problems effectively (SANC 2013).

Previous studies by Ali, Banan and Seraty (2015) showed that the lack of proper facilitation by professional nurses and clinical facilitators is a challenge during the facilitation of students (Ali et al. 2015). Furthermore, RNs are uncertain of their role as facilitators as there is no clarity as to who is accountable for the clinical accompaniment of student nurses (Rikhotso, Williams & De Wet, 2014; Kaphagawani & Useh, 2018).

**FIGURE 1:**
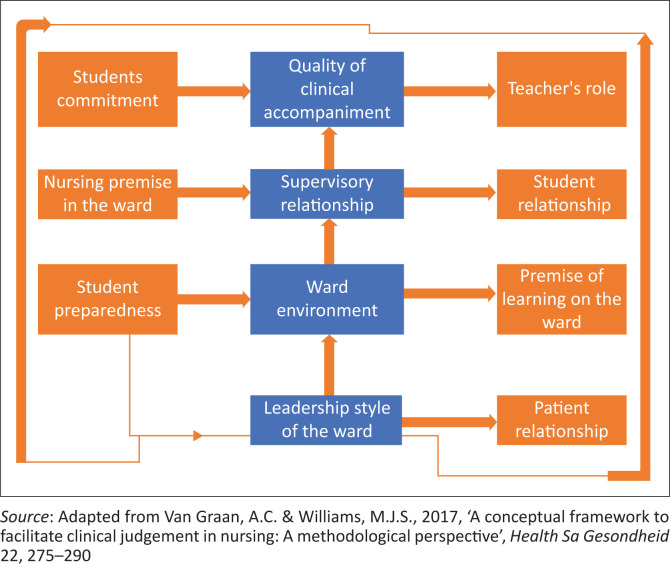
Conceptual framework to facilitate clinical judgement in nursing.

A study by Letswalo and Peu (2015) conducted in South Africa revealed that the RNs were not afforded sufficient time to facilitate students because of the pressure of other clinical activities and duties during the caring of the patients (Letswalo & Peu 2015). Moreover, a study by Motsilanyane (2015) conducted in South Africa indicated that in as much as clinical accompaniment is important to undergraduate students, there remains no clarity regarding who is accountable for the supervision of nursing students during their clinical practice. The scholar also maintains that the problem could be resolved by the placement of clinical facilitators at colleges and institutions to accompany students during the learning practice (Motsilanyane 2015). However, it does not relieve professional nurses from their teaching role. It is thus important to investigate the challenges that clinical accompaniment presents for the undergraduate nursing students. This study adopted the Van Graan and Williams’s (2017) conceptual model of teaching as it was found to facilitate clinical accompaniment and was appropriate to the study (Van Graan & Williams 2017). Figure 1 illustrates how the nursing students interact with the health personnel in the clinical learning practice in delivering services to the patients. The clinical learning of students involves the manager as the leader, ward environment, supervision of students by nurses, caring for the patients, teacher’s role and contribution to the quality of clinical accompaniment as indicated in Figure 1.

This study will help leaders in nursing schools make rules that can solve problems students face when they are in hospitals. Thus, policy makers will also be aided in developing clear guidelines that will address issues related to the health of the patients in South Africa and advance the country towards achieving Sustainable Development Goals (SDGs) 3 and 4. The SDG 4 goal refers to quality education, that is equitable quality education for all. The study findings will guide policymakers in acquiring empirical evidence to assist in developing standard operating procedures and manuals for nurses to contribute when providing care as well as teaching the nursing students (Newland 2017). Furthermore, through the implementation of the study results, nurses in South Africa and other sub-Saharan African countries will be able to provide and promote well-being for all ages, and most evidently nursing has a major role to play in relation to SDGs. Moreover, qualified nurses are responsible for education and regulation standards related to the profession, and for this reason, the study findings will assist in achieving SDG 4 (Quality education) for nursing students during their clinical accompaniment (Newland 2017).

This study aimed to determine challenges faced by the University of KwaZulu-Natal (UKZN) undergraduate student nurses during their accompaniment in the learning practice. The study objectives were to elucidate challenges encountered by UKZN student nurses during their clinical accompaniment and to compare the differences in student accompaniment challenges among first-, second-, and third-year students.

This study will help leaders in nursing schools make rules that can solve problems students face when they are in hospitals. Thus, policy makers will also be aided in developing clear guidelines that will address issues related to the health of the patients in South Africa and advance the country towards achieving Sustainable Development Goals (SDGs) 3 and 4. The SDG 4 goal is quality education, that is equitable quality education for all. The study findings will guide policymakers in acquiring empirical evidence to assist in developing standard operating procedures and manuals for nurses to contribute when providing care as well as teaching the nursing students (Newland 2017). Furthermore, through the implementation of the study results nurses in South Africa and other sub-Saharan African countries will be able to provide and promote well-being for all ages, and most evidently nursing has a major role to play in relation to SDGs. Moreover, qualified nurses are responsible for education and regulation standards related to the profession, and for this reason the study findings will assist in achieving SDG 4 (Quality education) for nursing students during their clinical accompaniment (Newland 2017).

This study aimed to determine challenges faced by UKZN undergraduate student nurses during their accompaniment in the learning practice. The study objectives were to elucidate challenges encountered by UKZN student nurses during their clinical accompaniment and to compare the differences in student accompaniment challenges amongst first-, second- and third-year students. This study will help nursing students learn better during their Clinical training.

This study wanted to find out what problems UKZN student nurses face while they are learning in a hospital. This study wanted to see what problems UKZN student nurses face when they are doing their practical training. It also wanted to compare the problems that first-year, second-year, and third-year students have.

Moreover, qualified nurses are responsible for education and regulation standards related to the profession, and for this reason the study findings will assist in achieving SDG 4 (Quality education) for nursing students during their clinical accompaniment (Newland, 2017).

## Methods

### Study design

An observational, cross-sectional study with an analytic component was executed.

### Study population

The student population at UKZN is approximately 45 000 with a growth rate in postgraduate enrolment of up to 30% of the total student enrolment (UKZN 2022). There are approximately 400 registered nursing students at UKZN, of which 245 are undergraduate students. The study participants were experienced in the clinical learning practice and were enrolled at UKZN. The participants in this study provided their written informed consent to freely participate in this research.

### Inclusion criteria

The eligibility of the student cohort to determine the participation criteria for the research project was carefully considered. As detailed earlier all the first-, second-, and third-year students at the UKZN were included in this study.

### Exclusion criteria

All UKZN students who met the needed criteria enrolled in this study except those who were not present during the data-collection process. University of KwaZulu-Natal students who did not belong to the School of Nursing and Public Health were not allowed to participate in this study. The nursing students who did not consent were excluded from participating in this study.

### Sampling strategy

All the undergraduate nursing students at UKZN were included using purposive sampling. Purposive sampling, also known as selective sampling, focuses on the characteristics of the population being studied with the aim of answering the research question. Purposive or homogeneous sampling was utilised to determine the sample as all the study participants shared the same commonalities in this study. Purposive sampling also saved time while collecting the data and the researcher was able to produce results representative of the specific population, that is, the nursing students (Suen, Huang & Lee 2014).

### Study setting

This study was conducted at the Howard College campus among the undergraduate nursing students enrolled at the University of KwaZulu-Natal.

### Data collection

The adapted five point-Likert scale questionnaire was utilised to determine challenges faced by the undergraduate student nurses during their clinical accompaniment (Chuan & Barnett, 2012). The researcher obtained written permission from Chuan and Barnett for the utilisation of their questionnaire in this study. The questionnaire comprised four sections, these being welcome and orientation; clinical accompaniment; support and opportunities for learning; learning environment. Section one of the questionnaire dealt with challenges relating to the welcome and orientation processes and comprised eight questions; section two comprised nine questions that measured clinical accompaniment; section three dealt with support and opportunities for learning and comprised six questions; and section four contained 26 questions measuring the clinical learning environment.

The study participants met at the UKZN Skills Laboratory where the researcher introduced the study and distributed the questionnaires, consent forms, and information sheets during the data collection. For this reason, it was easy for the participants to read and understand the contents of the distributed forms without requiring any assistance or translation. The questionnaires were distributed to the study participants on different days as movement was restricted because of the coronavirus disease 2019 (COVID-19) protocols in place at the time.

The study participants were allocated 30 min to respond to the given questionnaire. Participants were able to ask questions to clarify unclear aspects and responded to the questions individually without collaborating with their colleagues. To ensure their anonymity, no spaces for names or signatures were provided on the given questionnaires. Once the questionnaires were completed, the signed consent forms and information sheets were collected by the researcher and placed separately into sealed envelopes. The researcher used English as the preferred language for the questionnaires, consent forms, and the information sheets as it was both an official and international language.

### Data analysis

The structured questionnaire assisted the researcher to compute the exact percentage of responses from the participants who had experienced challenges in clinical accompaniment (Hall-Lord, Theander & Athlin 2013). The collected data were captured into the SPSS Statistics Package V28 and presented in the form of tables. Frequency distribution tables and proportions were used to summarise the study demographic characteristics and categorical variables within the study, while numerical factors were outlined utilising measures of central tendency (mean) and variability (standard deviation as well as interquartile range). One-way analysis of variance (ANOVA) was used to rule out differences between opinions of study participants regarding their challenges in clinical practice during clinical accompaniment.

### Ethical considerations

Ethics clearance was granted by the Department of Nursing, Humanities and Social Science Research and the Dean of UKZN School of Nursing and Public Health (BREC/00001617/2020).

### Reliability and validity measures

The questionnaire comprised questions that were relevant to the study to ensure validity in the proposed study project. The questionnaire was presented to the discipline of nursing and reviewed by one international and one local expert on clinical teaching for validity. Cronbach’s alpha was executed to determine reliability in this study to illustrate how consistent the given statements were in measuring the same construct (Cronbach 1951). A Cronbach’s alpha of 0.86 was obtained in this study.

## Review findings

### Demographics of the study participants

The data in Table 1 indicate that this study comprised first year (32.4%), second year (32.8%), and third year (34.9%) nursing students. The majority of the participants were female (73.9%). Most of the study participants were in the 18-to-25-year age group (97%) (see Table 1 which sets out the above demographic information in detail). The next section presents the challenges of the nursing students during clinical accompaniment as per the questionnaire sections 1 to 4.

**TABLE 1:** Demographics of study participants.

Characteristics	Frequencies	Percentage
**Age (years)**		
18–25	234	97.1
25–30	5	2.1
30 and above	2	0.8
Total	241	100
**Sex**		
Male	63	26.1
Female	178	73.9
Total	241	100
**Year of study**		
First-year	78	32.4
Second-year	79	32.8
Third-year	84	34.9
Total	241	100

#### Section 1: Welcome and orientation

The study findings as per Table 2 reveal that most of the participants (92.1%) who were orientated on clinical skills according to their level of study agreed with the findings. There was a high level of agreement (74.3%) with the statement that, when allocated to the CLE they were orientated to the duty roster. A total of 71.4% of the participants agreed they were allocated to different wards during clinical accompaniment. The majority (75.1%) agreed that during accompaniment in the CLE, they were assigned duties according to their level of training. In answer to the statement that they were treated as student nurses undergoing training at the clinical facility, 73.9% agreed with the statement (Table 2).

**TABLE 2:** Welcome and orientation.

Responses to the questionnaire:	Disagree		Neutral		Agree	
	*n*	%	*n*	%	*n*	%
Orientation of clinical skills done	9	3.7	10	4.1	222	92.1
All skills were performed	77	32.0	55	22.8	109	45.2
Students were orientated on tea and lunch times	87	36.1	30	12.4	124	51.5
Learners were orientated on duty roster	37	15.4	25	10.4	179	74.3
Students assigned to different wards	40	16.6	29	12.0	172	71.4
Duties assigned according to level of training	33	13.7	27	11.2	181	75.1
Learners compete for skills	84	34.9	49	20.3	108	44.8
Students treated as novice learners	33	13.7	30	12.4	178	73.9

*Source*: Adapted from Chuan, O.L. & Barnett, T., 2012, ‘Student, tutor, and staff nurse perceptions of the clinical learning environment’, *Nurse Education in Practice* 12, 192–197

#### Section 2: Clinical accompaniment

The results in Table 3 show that most of the participants (78.0%) agreed that they were provided with a clinical schedule on clinical accompaniment by the clinical facilitator. The majority of participants (76.3%) agreed that a reasonable and manageable number of students in wards promoted teaching. The participants (71%) agreed with the statement that they received constructive feedback from the clinical facilitator. Regarding the statement that they were provided with a clinical schedule, 78% supported the statement. The majority of the participants (69.3%) agreed that the clinical facilitator had a positive attitude towards students during their clinical accompaniment.

**TABLE 3:** Clinical accompaniment.

Responses to the questionnaire	Disagree		Neutral		Agree	
	*n*	%	*n*	%	*n*	%
I received clinical accompaniment from the clinical facilitator	40	16.6	36	14.9	165	68.5
I was provided with a clinical schedule on clinical accompaniment by the clinical facilitator	24	10.0	29	12.0	188	78.0
Received support visits from the clinical facilitator	68	28.2	54	22.4	119	49.4
Facilitator spent time with learners	76	31.5	38	15.8	127	52.7
I knew the clinical facilitator allocated to me on my clinical accompaniment	78	32.4	38	15.8	125	51.9
Facilitator portrays positive attitude towards learners	34	14.1	39	16.2	167	69.3
Feedback given to students	40	16.6	30	12.4	171	71.0
The number of nursing students in the clinical learning environment (CLE) promoted learning	18	7.5	39	16.2	184	76.3

*Source*: Adapted from Chuan, O.L. & Barnett, T., 2012, ‘Student, tutor, and staff nurse perceptions of the clinical learning environment’, *Nurse Education in Practice* 12, 192–197

#### Section 3: Support and opportunities for learning

Table 4 shows that the majority of the participants (90%) agreed that the ward should be considered as a positive learning environment. Regarding the statement that basic familiarisation was well-organised in the learning environment, 80.1% agreed. Most participants (79.7%) agreed that learning situations were meaningful during practice. Many of the participants (83.4%) were positive regarding the statement that there was a clear information flow relating to patients’ care. The majority (83.0%) agreed that documentation relating to nursing (e.g. nursing plans, daily recording of nursing procedures and so forth) was clear.

**TABLE 4:** Support and opportunities for learning.

Responses to the questionnaire:	Disagree		Neutral		Agree	
	*n*	%	*n*	%	*n*	%
Basic familiarisation was well organised in the learning environment	12	5.0	36	14.9	193	80.1
Learning situations on the ward were meaningful	19	7.9	30	12.4	192	79.7
There were multi-dimensional learning situations	9	3.7	46	19.1	186	77.2
Wards were considered important for teaching	9	3.7	14	5.8	217	90.0
There were clear patients care information	13	5.4	27	11.2	201	83.4
Clear documentation of nursing care plans	11	4.6	30	12.4	200	83.0

*Source*: Adapted from Chuan, O.L. & Barnett, T., 2012, ‘Student, tutor, and staff nurse perceptions of the clinical learning environment’, *Nurse Education in Practice* 12, 192–197

#### Section 4: The learning environment

Table 5 shows that students learned new skills as evidenced by 73% of participants who agreed with the statement. The study participants (79.3%) supported the statement that the clinical facilitator provided students with feedback. The findings also reveal that high quality of care was given to patients as 77.2% study participants supported this statement. In this study, it was found that students were regarded as learners rather than workers in the clinical practice as shown by 65.5% participants who agreed. Nurses showed positive attitudes towards students; 62.7% study participants supported this statement. Participants had competition while conducting practicals and 62.2% agreed with this statement. The majority (88.0%) of the participants agreed that student nurses assisted each other with their allocated tasks.

**TABLE 5:** The learning environment.

Responses to the questionnaire	Disagree		Neutral		Agree	
	*n*	%	*n*	%	*n*	%
Provision of feedback given to students	43	17.8	41	17.0	157	65.1
Nurses spend time on teaching	48	19.9	52	21.6	141	58.5
New skills were acquired through guidance	27	11.2	38	15.8	176	73.0
Nurses showed positive attitudes to students	41	17.0	49	20.3	151	62.7
Nurses could identify learners	62	25.7	65	27.0	114	47.3
Quality care provided to patients	13	5.4	42	17.4	186	77.2
Students were regarded as learners in practice	39	16.2	49	20.3	153	65.5
Facilitator motivates learners	8	3.3	33	13.7	200	83.0
Facilitator gives feedback	17	7.1	33	13.7	191	79.3
Students could approach facilitator	28	11.6	49	20.3	164	68.0
New skills were acquired through supervision	19	7.9	36	14.9	186	77.2
Facilitator was knowledgeable	8	3.3	14	5.8	219	90.9
Supervisor devotes time for students	33	13.7	50	20.7	158	65.6
Facilitators readily available	29	12.0	36	14.9	176	73.0
Enjoyed hospital practice	9	3.7	45	18.7	187	77.6
Looking forward to clinical practice	6	2.5	20	8.3	214	88.8
Students were motivated to learn	19	7.9	32	13.3	190	78.8
Little assistance offered	83	34.4	49	20.3	109	45.2
Stressed by amount of work to be done on the ward	52	21.6	59	24.5	130	53.9
Theory learned contradicts the actual practice	59	24.5	54	22.4	128	53.1
Students assigned work with little guidance	80	33.2	51	21.2	110	45.6
Students compete to practise skills	48	19.9	43	17.8	150	62.2
Reinforcement of theory learned	15	6.2	43	17.8	183	75.9
Learners taken as ward staff	14	5.8	52	21.6	175	72.6
Student are motivated to ask for clarity	12	5.0	25	10.4	204	84.6
Students help each other	7	2.9	22	9.1	212	88.0

*Source*: Adapted from Chuan, O.L. & Barnett, T., 2012, ‘Student, tutor and staff nurse perceptions of the clinical learning environment’, *Nurse Education in Practice* 12(4), 192–197. https://doi.org/10.1016/j.nepr.2012.01.003

### Determining the differences in challenges among the participants (ANOVA descriptive results)

One-way analysis of variance was used to ascertain if there were remarkable differences between first, second, and third year groups regarding clinical accompaniment challenges. The results in Table 6 indicated that clinically there were differences as evidenced in the different mean scores. The mean scores for the first-years (M = 185.4, s.d. = 19.2) were lower than for the second-year students (M = 188.0, s.d. = 25.1) and for the third-years (M = 184.6, s.d. = 22.0). However, there were no significant differences among study participants, *p* = 0.592.

**TABLE 6:** Determining the differences in challenges.

Level of study	*N*	Mean scores	95% confidence		Standard deviation	*p*
			Lower-bound	Upper-bound		
First year	78	185.4	181.1	189.7	19.2	**0.592**
Second year	79	188.0	182.4	193.7	25.1	
Third year	84	184.6	179.8	189.4	22.0	

**Total**	**241**	**185.9**	**183.2**	**188.8**	**22.2**	

Note: *p*-value of 0.592 indicates significance difference of the mean scores of challenges faced by the 3 study levels of students.

## Discussion

The study assessed the challenges of clinical accompaniment for undergraduate nursing students at UKZN in line with the stated objectives of the study. Overall, this study indicated that most of the study participants agreed that challenges in learning practice do exist. A detailed discussion was presented under five main categories, namely, socio-demographic findings, welcome and orientation, clinical accompaniment, support and opportunities for learning, and CLE.

### Socio-demographic findings

In this study, socio-demographic characteristics of participants included sex, age as well as year of study and were discoursed on in terms of categories consisting of 18 years–25 years 97% (*n* = 234) followed by 25 years–30 years 2% (*n* = 5) and the final category between 30 years and above 0.8% (*n* = 2). Most of the study participants were females 73.9% (*n* = 178) while male participants constituted only 26.1% (*n* = 63) of the cohort. A total of 32.4% (*n* = 78) of the participants represented first-years 32.8% (*n* = 79), second-years, and 34.9% (*n* = 84) third-years.

The age group 18 years–25 years (97%; *n* = 234) comprised more participants than the other categories. Similar to the study findings, Flott and Linden (2016) revealed that the category 18 years–25 years (94%) represented more participants. The age group (18 years–25 years) represented more participants as students preferred nursing as a career before or between the ages of 20 years and 30 years of age (Stanley et al. 2016).

The research findings in this current study show that there were almost an equal proportion of challenges among study participants regarding issues in clinical accompaniment. This correlates with previous studies whereby scholars such as Noordien, Hoffman and Julie (2020) reported fair distribution among 1st year (25%), 2nd year (27%), and 3rd year (20%) study participants (Noordien et al. 2020). The rationale for the equal proportion among these categories of nursing students could have resulted from the academic enrolment or students withdrawing from the course (Noordien et al. 2020).

### Welcome and orientation

The research findings in this study reveal that the welcoming and orientation of nursing students by their clinical facilitators was of paramount importance. This was evidenced in the fact that 92% of the participants (*n* = 222) agreed that they were orientated on clinical skills related to their individual levels of study. In addition, 74.3% of the participants (*n* = 179) indicated that they were orientated on the duty roster. A total of 71.4% of the participants (*n* = 172) mentioned that when allocated to different wards, they were given sufficient time to orientate themselves which promoted learning, as shown in Table 2. Similar to the study findings, Janula Raju, Megahed and Chithra (2017) revealed that orientation programmes are considered key to a student’s career success. Hassan, Ibrahim and Hussain (2017) concur, stating that nursing students gain significant confidence in clinical skills through planned and effective orientation, which in turn results in job satisfaction. The orientation programme is regarded as a continuous process that can create a healthy learning environment for student nurses in the clinical practice (Hassan et al. 2017). Thus, if carried out accordingly, the set learning objectives as per the learning institutions can be achieved. It is also important to clarify and arrange tasks for the nursing students during their orientation process (Flott & Linden 2016).

Moreover, similar to study findings, orientation of nursing students is fundamental during the nursing practice. Flott and Linden (2016) maintain that practical clinical tasks influence learning outcomes for students; hence, student nurses should participate during welcoming orientation programmes when allocated to clinical learning practice.

Furthermore, this study found that during clinical accompaniment most study participants experienced rivalry from other nursing institution as evidenced by 44.8% of the participants (*n* = 108) indicated in Table 3. This rivalry may impact negatively on their welcome and orientation. Similar to the study findings, Mbakaya et al. (2020) revealed that overcrowding in the clinical practice influenced students support which in turn resulted in conflict, tension, and competing for learning opportunities. These negative conditions result in a lack of fulfilling the required competencies and jeopardise the care rendered to the patients during the clinical accompaniment.

This study findings and those of Mbakaya et al. (2020) regarding rivalry are also in line with the study findings of Allari & Farag (2017) which indicated that during clinical accompaniment students struggle to access learning opportunities because of overcrowding of learners being assigned to one department (Ahmadi et al. 2018). These findings are similar to those of Kaphagawani and Useh (2013), who found that wards were overcrowded and therefore it negatively impacted on the students’ learning practice (Kaphagawani & Useh, 2013b). The findings of Truong (2015), on the other hand, show that wards with a reasonable number of students and clinical facilitators were able to successfully respond to the students’ learning needs compared to wards with large numbers of nursing students (Truong 2015).

### Clinical accompaniment

From the findings of this study, it was established that it is important for students to be accompanied by facilitators while in clinical practice; this was supported by majority of study participants (71%; *n* = 171) who indicated that nurses and facilitators provided constructive feedback. Additionally, 76.3% (*n* = 184) of the participants indicated that during clinical accompaniment, a reasonable number of students allocated per ward enabled learning to take place without competing for procedures and practicals. These findings correlate with other scholars such as Koharchik and Redding (2016) who concurred that providing feedback was a crucial component in the clinical teaching and learning process for nursing students. Thus, all the leaders of the institutions of higher learning should regard providing positive feedback as a critical aspect of the institution’s teaching culture (Koharchik & Redding 2016).

Similar to the study findings of this research, studies indicated that constructive feedback provides students with the opportunity to build necessary sensitivity and provide insight into the values of those whom they interact with, as nursing students do not only interact with their clinical facilitators but also with other health care personnel (Allari & Farag 2017).

Interestingly, although previous studies reported the importance of feedback, the study findings of Panneerselvam (2018) indicated that the provision of negative feedback particularly when added to the lack in communication, impacted negatively on the learning of nursing students. Therefore, students can learn efficiently if they are aware of their academic performance in the clinical learning practice, as this encourages them (Panneerselvam 2018).

The study also found that during the clinical accompaniment of nursing students, support visits from the clinical facilitator was an essential component in the clinical area. This was indicated by 49.4% (*n* = 119) of the participants who agreed with this statement; however, there were a few disagreements (28.2% *n* = 68). Additionally, in the matter of facilitators spending time with nursing students, 52% (*n* = 127) of the participants were positive. A total of 31.5% (76) disagreed that clinical facilitators spent time in the CLE (see Table 4). Allari and Farag (2017) stated that support visits should be considered as an important aspect in the learning and teaching environment of the students particularly as the availability and presence of clinical facilitators in the clinical practice provide opportunities for teaching, guiding, and demonstrating the procedures to the students. This will also assist the facilitators to evaluate the students’ performance and re-assess the areas that require improvement (Allari & Farag, 2017; Salamonson et al., 2015). The study by Zulkosky, Minchhoff and Dommel (2021), similarly resonates with the importance for academic support visits during clinical accompaniment (Zulkosky et al. 2021).

A study by Bvumbwe, Malema and Chipeta (2015) showed that some institutions offer little or no support to student nurses during clinical accompaniment (Bvumbwe et al., 2015). For this reason, nursing students became anxious and disorganised, and as a result, the relationship between students and nurse educators was negatively affected, which in turn hampering the clinical learning process. Therefore, the clinical facilitator’s role extends beyond classrooms and simulation laboratories as their role is crucial in bridging the gap between theory and practice.

Moreover, research revealed that time allocated during clinical accompaniment was insufficient for students to acquire necessary skills and competencies. As a result, students were not able to build a sound rapport with the clinical staff as well as with patients (Hatupopi & Nuuyoma 2019). Therefore, it is important for students to be given adequate time in each department to become acquainted and well-equipped with the necessary skills and knowledge (Hatupopi & Nuuyoma, 2019).

### Support and opportunities for learning

According to the findings in this study, support and opportunities for learning are important in the learning environment. This was proven by the fact that most of the study participants (90%; *n* = 217) agreed that the ward could be considered as an extended and productive learning environment as there was a clear information flow regarding patient care (83.4%; *n* = 201). The literature by Van Graan and Williams (2017) supports the notion that ward atmosphere was conducive to learning; participants were motivated and gratified with the experience.

The study findings of Walker et al. (2014) indicated that for learning to take place, there must be experienced practitioners available in the CLE (Walker et al. 2014). The experienced instructors assist students to improve on their skills, knowledge, professional socialisation, and confidence. This study concurs with these findings and endorses the reason of providing support to nursing students through the clinical learning practice.

The results in this study also indicated positive attitudes in other areas of support and opportunities for learning. These included attributes such as sufficient, meaningful learning (79.7%; *n* = 192), multi-dimensional learning conditions (77.7%; *n* = 186), and documentation of nursing (83%; *n* = 200). This study is consistent with the views of Labrague et al. (2015), that the role of the clinical facilitator is to ease the mechanisms that will increase critical thinking, problem solving skills, and caring for patients in a safe environment (Labrague et al. 2015).

### Clinical learning

This study found that care delivered to patients in the CLE was of a high standard. This was evidenced by 77.2% (*n* = 186) of the participants who felt positive about this aspect. These findings agree with the findings of Papastavrou et al. (2016), which indicated that providing high-quality care to the patients is a major component of the CLE (Papastavrou et al. 2016). Previous studies by Lawal et al. (2016) indicated that nursing education stakeholders emphasised that the training of dedicated and knowledgeable nurses could only be achieved in a learning practice where the patients are provided with excellent services. This study concurs as the findings indicate that 77.2% of the study participants agree that patients received good care during their clinical accompaniment (Lawal et al. 2016).

This study revealed that during their clinical accompaniment, nursing students were regarded as ward teams. This was substantiated by 72.6% (*n* = 175) of the study participants who supported this statement. Similarly, Mbakaya et al. (2020) stated that students acquire skills effectively in an environment that promotes learning, by motivating and assisting them as well as by acknowledging them as part of the profession. The study by Msiska, Smith and Fawcett (2014) reported that students were actually considered as working personnel rather than learners. When the learning environment is not conducive to teaching, students are exposed to vulnerabilities such as insecurity and anxiety (Msiska et al. 2014).

It also emerged from this study that the clinical facilitator was regarded as an efficient role model. This was supported by the high level of agreement among the participants (83%; *n* = 200) as shown in Table 6. Similar to the study findings, Van Merriënboer and Kirschmer (2017) stated that role modelling requires that the clinical facilitator demonstrates the skills required so as to allow the students the opportunity to observe and later carry out such skills themselves. The findings of Sabog, Caranto and David (2015) concur that role modelling and competency are fundamental to the teaching of nursing students as they enable the clinical facilitator to incorporate theory into the real world of nursing (Sabog et al. 2015).

The study findings indicated that the nurses in the learning environment generally had a positive attitude toward nursing students by devoting enough time to teach and guide the students (65.6%, *n* = 158; 73%, *n* = 176). The study by Allari & Farag (2017) reported that for nursing students to attain outcomes, nurses should provide opportunities for learning. The study by Dehghani, Salsali and Cheraghi (2016) indicated that challenges such as staff being unapproachable, unfriendly, and displaying a negative attitude in the clinical practice environment will adversely influence the students (Dehghani et al. 2016). Thus, denying student nurses the opportunity to learn and to be guided results in poor relationships and as a result students become distracted and discouraged leading to a poor acquisition of knowledge.

Other challenges regarding clinical accompaniment were difficulties in finding help; 45.2% (*n* = 109) of the participants agreed and 34.4% (*n* = 83) disagreed, being stressed because of the workload. A total of 53.9% (*n* = 130) of the participants agreed, 21.6% (*n* = 52) disagreed and responsibilities were allocated without supervision; 45.6% (*n* = 110) of the participants agreed and 33.2% (*n* = 80) disagreed. The study by Tiwaken, Caranto and David (2015) similarly reported that undergraduate nursing students having difficulties in finding assistance when needed experienced stress and the inability to learn and focus effectively.

According to Moon, Mclnnes and Melton (2015), it was reported that nursing students were dissatisfied with the CLE as oftentimes students were not able to perform some of their nursing duties because of a lack of equipment (Moon et al. 2015). These findings were corroborated by Rikhotso et al. (2014), as their study findings confirmed that the learning practice could not support learning because of outdated and inadequate equipment in the hospitals, as well as the lack of staff to offer guidance and support to the students. In contrast, in a study conducted by Webster et al. (2018), it was reported that nursing students enjoyed a wide range of opportunities for learning, such as learning about professional roles, reflecting on personal skills, performance, and so forth. For example, ‘nursing students were identified by medical students as being better at drawing up drugs, setting up intravenous drips, and keeping records’ (Webster et al. 2018:160).

In comparing the differences in challenges in the clinical accompaniment environment, an ANOVA was conducted among the groups of first, second-, and third-year participants. This research found no statistical differences (*p* = 0.592) regarding the challenges faced by the nursing students that influenced their learning. However, the findings of this study also indicated that there were in fact some differences in the challenges in clinical accompaniment. This was evidenced in the difference of the mean score. For example, the first-year mean scores (M = 185.4, s.d. = 19.2) were lower than for the second-year students (M = 188.0, s.d. = 25.1) and for the third-year students (M = 184.6, s.d. = 22.0), as indicated in Table 6. This implies that the first-year students experienced less challenges when compared to the second-year and third-year students. The rationale for these differences in challenges is that, during the initial exposure to clinical practice, first-year students focus more on adapting to the clinical environment and professional activities, whereas second and third-year students are already working towards achieving the programme outcomes.

Similar to the findings of this study, Gilmour et al. (2013) reported that experiences at the beginning of the practice periods (first-year students) could vary and influence the students’ expectations and self-esteem; hence, they sometimes felt distracted and anxious prior to their first exposure to the CLE (Gilmour et al. 2013).

Moreover, this study findings are also consistent with previous studies which found that first-year students faced challenges in the clinical practice as a result of being new to the environment. For instance, novice students were unfamiliar with the team, for example doctors and nurses as well as the patients; hence sometimes the patients preferred to be attended to by the experienced and qualified nurses rather than accept the services offered by the students, as some patients believed that students lacked adequate experience or knowledge of the clinical practice (Pouralizadeh et al. 2017). The findings of Gilmour et al. (2013) and Walker et al. (2014) indicated that the beginning of the clinical practice represented a stressful and troublesome phase for the students. Students in the CLE were often afraid of making mistakes when performing procedures on the patients and/or harming the patients because of their lack of knowledge and experience.

The study by Wawire et al. (2014) discovered significant differences among nursing students in the clinical learning practice pertaining to the satisfaction in the CLE versus the ideal clinical learning practice (Wawire et al. 2014). According to Rajeswaran (2017), nursing students in their second and third year of study experienced more challenges such as stress compared to the first-year students. The reason for such differences was that the second-and third-years were anxious that they might injure the patients, lack in experience in carrying out certain nursing procedures, as well as struggle with the study load (Rajeswaran, 2017; Kamphinda & Chilemba, 2019).

A study by Ahmadi et al. (2018), instead of focusing on the level or year of study, reported that the age of the student was also linked to the challenges of accompaniment in the CLE. Senior students were often more challenged in performing nursing duties compared to the novices. This is perhaps because of the changing objectives and expectations in the institutions as one advances to the next level and sometimes because undergraduate nursing students have less or no clinical experience compared to students who are enrolled in the Bachelor of Advanced Nursing Programme and who already had some exposure, had been orientated, and were already working as professional nurses (Ahmadi et al., 2018; Awuah-Peasah, Sarfo & Asamoah, 2013).

In another study by Cremonini et al. (2015), it was reported that there were differences among the groups of first-year, second-year, and third-year nursing students in the learning practice (Cremonini et al. 2015). Their study findings indicated significant differences among these groups (*p* = 0.46) in relation to the challenge of supervisory relationships. Considering the challenges of clinical accompaniment, the first year nursing students differed considerably from the second year and third year students in this study. Moreover, in line with these study findings, Sabog et al. (2015) reported a significant difference in the challenges of clinical instructor relationships among the undergraduates and senior nursing students (Sabog et al. 2015).

## Study limitations

The study was limited to one institution; thus results cannot apply to other institutions. However, a complementary study should be conducted at other institutions in KwaZulu-Natal in order to determine possible similarities and differences regarding accompaniment challenges for undergraduate nursing students. This study also relied on self-reporting data which was considered to be another limitation to this study because the participants may have over-rated or under-rated their views in response to the questionnaire.

The researcher highlighted the strengths of quantitative research in this study. In quantitative research, the findings can be statistically validated and the collected data are consistent, precise, reliable, and easy to analyse (Sugiantari, Sari & Januraga 2018).

### Recommendations

A quantitative study incorporating more institutions of higher learning in KwaZulu-Natal (KZN) should be conducted in order to determine the similarities and differences from a wider perspective regarding the challenges of clinical accompaniment experienced by students.

## Nursing practice and education

Nursing institutions should regularly update the curriculum for teaching so that the content remains in line with current practices in the CLE. Professional nurses should undergo refresher training courses as this will further assist them in providing adequate guidance and supervision. Clinical facilitators should ensure their availability in the clinical learning practice as this will assist undergraduate nursing students to obtain the necessary clarifications when and where required. The institutions should implement strict rules against students who do not attend clinical practice as per the institution’s rules for clinical accompaniment.

## Conclusion

In summary, clinical accompaniment is a major component in nursing that enables students to understand clinical practice and makes it easier for them to acquire the necessary practical knowledge during their clinical accompaniment. The challenges inherent in clinical accompaniment were discussed in line with the study objectives under the following headings: socio-demographic findings, welcome and orientation, clinical accompaniment, support and opportunities for learning, as well as the CLE. Lastly, the various challenges present in clinical accompaniment among first year, second year, and third year students were discussed in this study.
